# Recipe for the Poor

**Published:** 1855-02

**Authors:** 


### RECIPE FOR THE POOR.

Do not give a penny to a beggar, to a street sweeping child, or to a young woman at your door, with two babies exactly not alike, *nor to any dirty person;* I never do; deserving poverty is not dirty; if but a rag to wear, there is mark of care about that rag. Shut up all the soup-shops, make no more calico dresses to wear once, and then send to Mr. Pease for the poor, whose self-respect is never increased by wearing the cast-off clothing of another; or receiving a penny or a dinner as alms. No! that is not the way, but collect every dollar possible, raise it to tens of thousands, open your heart wide for the poor, and send every mother's son and daughter of them towards Nebraska, through New York, Ohio, Indiana, Illinois and Missouri, and spill them out along the road, as I have seen the London wagons spill out the letter carriers along their route, the one nearest the entrance getting out first, so that the vehicle need not stop a moment. I know from having travelled along the road, that tens of thousands of laborers, men and women, boys and girls, are needed by Western farmers, who would be but too glad to get them for their board and clothing; and would even give fair wages besides.

Humanitarians! You are on the wrong track with your soup-shops and your alms-houses. You pay a premium on loafering, and men soon get to like it; you buy away their self-respect, and the feeling of independence, which makes all the difference between a man and a human thing, and all the while, taking the flattering unction to your souls, you are doing God service. Help your fellow mortal to help himself, and be a man, not a loafer, and angels will smile.

